# Discrimination of Rheumatoid and Psoriatic Arthritis Based on Raman and NIR Spectra Using Machine-Learning Algorithms

**DOI:** 10.3390/molecules30234513

**Published:** 2025-11-22

**Authors:** Przemysław Cuprych, Izabela Kokot, Roman Szostak, Ewa Maria Kratz, Sylwester Mazurek

**Affiliations:** 1 Department of Chemistry, University of Wroclaw, F. Joliot-Curie, 50-383 Wroclaw, Poland; przemyslaw.cuprych@uwr.edu.pl; 2 Department of Laboratory Diagnostics, Division of Laboratory Diagnostics, Faculty of Pharmacy, Wroclaw Medical University, Borowska Street 211A, 50-556 Wroclaw, Poland; izabela.kokot@umw.edu.pl

**Keywords:** rheumatoid arthritis, psoriatic arthritis, Raman spectroscopy, NIR spectroscopy, machine learning, discriminant analysis

## Abstract

Rheumatoid arthritis (RA) and psoriatic arthritis (PsA) are chronic autoimmune diseases. They share similar symptoms. The lack of specific markers can lead to misdiagnosis. Using spectroscopic information on the chemical composition of body fluids can effectively differentiate these diseases. The discriminant analysis results are presented based on Raman and near-infrared (NIR) spectra of freeze-dried blood sera. The performance of partial least squares discriminant analysis (PLS-DA) and counter-propagation artificial neural network (CP-ANN) techniques in differentiation between RA (*n* = 30) and PsA (*n* = 24) patients and healthy controls (HC, *n* = 15) were compared. Samples were divided into calibration and validation sets using a Kennard–Stone algorithm; approximately 1/3 of the samples were selected for external validation. The PLS-DA and CP-ANN models built based on spectral features selected using the interval partial least squares (iPLS) algorithm resulted in an overall accuracy (OA) for test samples prediction in the 81.3–93.8% range. Hybrid models elaborated using a combination of selected biochemical parameters of blood serum and spectral variables were characterized by OA values from 87.5 to 93.8%. The obtained results confirm that vibrational spectroscopy and chemometric modeling enable discrimination of these two difficult-to-diagnose diseases on the basis of spectral data of the dried blood serum.

## 1. Introduction

Rheumatoid arthritis (RA) and psoriatic arthritis (PsA) are chronic autoimmune diseases that primarily attack the musculoskeletal system. The acute course of both of these conditions involves numerous extra-articular lesions in several vital organs. RA is characterized by symmetrical inflammation of the joints, degradation of articular cartilage, and bone epiphyses, and the most common symptoms include pain, stiffness, sensitivity to joint pressure, swelling resulting from synovial membrane proliferation, joint fluid exudation, the occurrence of rheumatoid nodules in the elbow and hand joints, and loss of the ability to perform a full range of motion in the affected joint. Extra-articular changes that occur in severe RA include valve damage and pericarditis of the heart, pleuritis, interstitial pneumonia, and numerous eye, kidney, and nervous system diseases. In the course of PsA, inflammation of the axial joints, peripheral joints, tendon attachments of the spine, and skin and nail changes resulting from the psoriatic nature of the disease are typical. In 70% of cases, PsA occurs in an asymmetric multi-joint form, in which the disease affected joints in various locations on both sides of the body. In some patients (up to 50%), the disease progresses in a symmetrical form, in which the disease affects joints in a similar way to RA. The remaining cases are divided into the axial form, which asymmetrically affects joints of the spine and the sacroiliac joint, the form with predominant inflammation of the distal interphalangeal joints, and the mutilating form, in which the joints are significantly deformed. The pathogenesis of the two diseases in question is not yet well understood. In the case of RA, it is mediated by anti-citrullinated protein antibodies, which, according to some theories, arise as a result of pneumonia occurring even before joint symptoms, whereas in the case of PsA, there are indications linking the genesis of the disease to intestinal dysbiosis. Inflammatory reactions arising in the course of RA and PsA are triggered by a number of cytokines. In the course of RA, these include the interleukins IL-1, IL-6, IL-22, and IL-23, the chemokines CXCL11 and CXCL13, and TNF-α, whereas in the course of PsA, the interleukins IL-17, IL-22, IL-23, IL-1β, IL-6, interferon-γ, and TNF-α are responsible for inflammation [1].

An unambiguous diagnosis of RA and PsA is still difficult due to the diseases’ similar nature and the lack of specific markers. Diagnosis is usually based on medical history, the values of the selected blood parameters, such as C-reactive protein and erythrocyte sedimentation rate, and the use of X-ray and ultrasound imaging techniques. The American College of Rheumatology and European League Against Rheumatism (ACR-EULAR) and the Classification Criteria for Psoriatic Arthritis (CASPAR) classification criteria are used to support RA and PsA diagnosis, respectively [2,3].

Despite significant progress in medicine, modern diagnostics still faces many challenges. An interview, determination of biochemical parameters of body fluids, or the use of imaging techniques is not always sufficient to accurately diagnose a given disease. New tools and techniques are constantly being sought to improve the diagnostic process, which is particularly important in the case of diseases with nonobvious symptoms. The use of vibrational spectroscopy is becoming increasingly common in diagnosing, differentiating disease states, and monitoring the stage of disease development. IR and Raman spectra of biological material include information about its biochemical composition. Molecules such as proteins, lipids, amino acids, and carbohydrates have characteristic representations in vibrational spectra; therefore, the changes in the body fluids’ or tissues’ molecular composition caused by disease or inflammatory state are reflected in the spectra. The numerous advantages of vibrational spectroscopy, i.e., the short time required to obtain a spectrum, low cost of measurement compared to immunological tests, high repeatability of results, the fact that the sample is not destroyed during measurements, and the possibility of recording spectra of samples in various physical forms, enable the use of various vibrational spectroscopy techniques in the diagnostics of autoimmune, oncological, neurological, dermatological, gynecological, respiratory, and gastrointestinal diseases [4,5,6].

So far, there are very few examples in the literature on diagnosing RA based on the vibrational spectra. Using FT-IR spectra of sera, Staib et al. [7] developed a linear discriminant model to distinguish patients suffering from RA from healthy controls with sensitivity and specificity ranging from 84 to 88%. Based on Raman spectra of serum lyophilizates, Carvalho et al. [8] developed a Linear Discriminant Analysis (LDA) model for RA characterized by higher accuracy than that found for a model constructed using rheumatoid factor (RF) and C-reactive protein (CRP) parameters. Canvin et al. [9] used a fiber-optic probe to collect near-infrared (NIR) spectra of the hand joints of patients in the early and late stages of the disease, and the LDA allowed for distinguishing between spectra of healthy and disease-affected joints with an overall accuracy of 74%.

There are no reports of other scientific groups except [10] on PsA diagnosis based on vibrational spectra, and no attempts have been made to differentiate patients with RA and PsA utilizing Raman or NIR spectra of body fluids until now. In our previous studies, we have demonstrated that combining selected biochemical parameters of blood serum with ATR-FTIR spectra results in an improvement of the obtained classifiers [10]. Here, we report the results of discriminant modeling based on NIR and Raman spectra of serum lyophilizates originating from RA and PsA patients. The efficiency of discriminant models built based on the selected ranges of these two types of spectra with those constructed applying combined biochemical and spectral data were compared. Despite their different etiopathogenesis, RA and PsA show very similar symptoms, hence the need to develop a methodology allowing for fast differentiation of these two inflammatory conditions.

## 2. Results and Discussion

### 2.1. Spectra of Serum Lyophilizates

**Figure 1:**
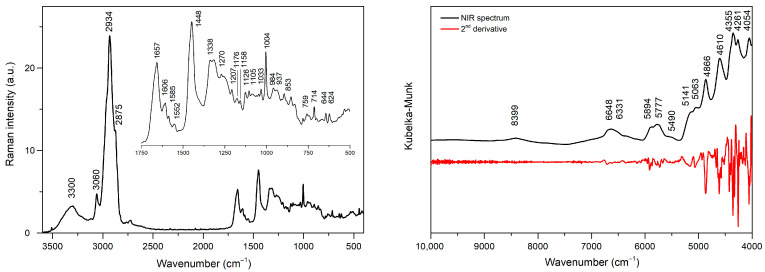
Raman (**left**) and NIR (**right**) spectrum of serum lyophilizates.

An average Raman spectrum of the studied samples is shown in Figure 1. In the 3000–3500 cm^−1^ range, a broad band with a maximum at 3300 cm^−1^ originating from the N-H and O-H stretching vibrations is visible. The band located at 3060 cm^−1^ can be attributed to the ν(C-H) vibrations of aromatic moieties. The most intense band present at 2934 cm^−1^ originates from the asymmetric vibration of the C-H bond whereas the one at 2875 cm^−1^ originates from the ν_s_(C-H) vibration. At 1657 cm^−1^, an amide I band contribution is visible, which mainly corresponds to the C=O stretching vibration in proteins’ peptide bonds. Signals at 1606 cm^−1^ and 1585 cm^−1^ can be associated with the ν(C=C) vibration of aromatic rings of tyrosine and phenylalanine. The band appearing at 1552 cm^−1^ originates from ν(C=C) vibration in tryptophan.

A relatively intense spectral feature found at 1448 cm^−1^ corresponds to the C-H deformation vibration of the CH_2_ group. The signal at 1338 can be attributed to the presence of DNA purine bases—adenine and guanine. At 1270 cm^−1^, an amide III band occurs, resulting from the combination of the stretching C-N and deformation vibrations of the N-H bond. The band at 1207 cm^−1^ is associated with the C-C bond stretching vibration in tryptophan and phenylalanine. At 1176 cm^−1^, a band corresponding to the deformation vibration of the C-H bond in the aromatic ring of tyrosine is present. The signal appearing at 1158 cm^−1^ can be associated with the stretching vibrations of the C−C and C−N bonds present in proteins. The C-N bond stretching vibrations is also responsible for a band occurring at 1126 cm^−1^. At 1105 cm^−1^, there is a signal associated C-N stretching vibrations, while bands located at 1033 cm^−1^ and 1004 cm^−1^ correspond to the benzene ring vibrations in phenylalanine. The band appearing at 937 cm^−1^ comes from the C-C stretching vibration in the α-helix structure of proteins, the C-C stretching backbone vibration is responsible for the signal at 894 cm^−1^, while the bands located at 853 cm^−1^ and 759 cm^−1^ can be attributed to the ring breathing mode in tyrosine and tryptophan molecules, respectively. A band at 714 cm^−1^ occurs due to the presence of polysaccharides, whereas the bands at 644 cm^−1^ and 624 cm^−1^ can be assigned to the C-C twisting vibrations in tyrosine and phenylalanine, respectively. An assignment of the most characteristic bands present in the Raman spectrum is summarized in Table 1.

The difference spectra (Figure S1 in the Supplementary Material) and spectral regions selected by the iPLS algorithm (Table S1 in the Supplementary Material) do not show clear correlation between changes in certain bands and disease states. The main differences are seen in the C-H stretching, C-H deformation and amide vibration regions. Alterations in these molecules may be connected to the inflammatory state of the diseased individuals.

The average NIR spectrum of serum lyophilizates and plot of the second derivative of the spectrum is shown in Figure 1. In comparison to Raman data, the NIR spectrum is less characteristic due to the presence of broad bands resulting from the overlap of overtones and combinations of fundamental vibrations. A band of moderate intensity located at 8399 cm^−1^ (1191 nm) can be assigned to the second overtone of C-H stretching vibrations. A broad band with a maximum at 6648 cm^−1^ (1504 nm) can be attributed to the first overtone of the stretching vibrations of the N-H and O-H bonds, while a doublet with maxima at 5893 cm^−1^ and 5777 cm^−1^ (1697 nm and 1731 nm) is associated with the first overtone of the asymmetric and symmetric vibrations of the C-H bond in the methylene groups, respectively. The presence of water in the analyzed samples manifests itself by combination bands of the O-H group with maxima at 5141 cm^−1^ and 5063 cm^−1^ (1945 nm and 1975 nm). The maxima found at 4866 cm^−1^ and 4610 cm^−1^ (2055 nm and 2169 nm) occur due to the combination vibrations of N-H. Three bands, visible at 4355 cm^−1^, 4261 cm^−1^, and 4054 cm^−1^ (2296 nm, 2347 nm, and 2467 nm), originate from the C-H combination vibrations. Tentative band assignment in the NIR spectrum of blood serum lyophilizates is summarized in Table 2.

### 2.2. Discriminant Analysis

Vibrational spectra of samples collected from the three studied groups of donors were very similar. Figures S1 and S2 in the Supplementary Materials show average Raman and NIR spectra of serum lyophilizates for the RA and PsA patients and the control group. In the entire spectral range, the intensities in the difference spectra were lower than the standard deviation of the mean signals for the studied group of objects, which indicates that it is not trivial to distinguish between RA and PsA samples based on visual evaluation of the spectra.

#### 2.2.1. Selection of Spectral Features

Selection of variables with the highest discriminative potential enables building efficient classifiers. In the case of spectral data, a large number of features and their combinations needed to be checked. In the preliminary step, PLS-DA models were constructed based on raw Raman and NIR spectra. To improve the classifiers’ discrimination ability, PLS loadings, variability importance projection (VIP), and selectivity ratio plots were used for optimization of the spectral ranges used; however, models constructed in this way were of moderate quality, with overall accuracy (OA) values for training and testing sets below 70%. An improvement of the models’ quality was obtained when spectral markers were selected using the iPLS algorithm. To determine the optimal number and width of the spectral windows used by iPLS, a screening procedure was performed for both types of experimental data. Overall accuracy for the test set prediction was chosen as a basic criterion for elaborated PLS-DA models.

**Figure 2:**
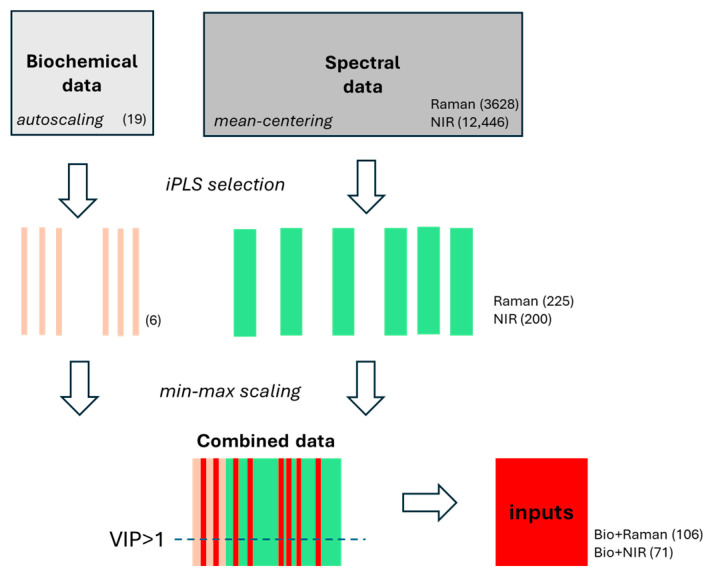
Scheme of data processing for PLS-DA and CP-ANN modeling; in parentheses, the number of variables is shown; the dashed line indicates the selection of variables for which VIP scores are greater than one.

For Raman spectra of serum samples originating from the three groups of donors, the number of iPLS intervals ranged from 10 to 20, and the width varied from 10 to 20 points, corresponding to the 8.7 and 18.3 cm^−1^ ranges of spectra, respectively. The highest OA value was obtained for the model including 225 features, from 15 intervals of 15 data points each (Tables S1 and S2 in Supplementary Material). A general scheme of data pre-treatment and selection utilized in discriminant analysis is shown in Figure 2.

It should be noted that Raman spectra of body fluids are characterized by rather moderate signal-to-noise ratio (S/N) values, so the presence of spectral noise can hamper the selection of spectral markers.

NIR spectra of lyophilizates can be influenced by the presence of water, which absorbs radiation in this range of electromagnetic radiation. Water signals in the 5000–5300 cm^−1^ and 6800–7200 cm^−1^ ranges, originating from ν_as_ + δ and ν_as_ + ν_s_ vibrations of O-H groups, can be observed. Despite identical storage and processing conditions, even minute differences in sample moisture can result in an undesirable variability that may affect the selection of the spectral ranges allowing for differentiation between RA and PsA patients. To improve the spectral resolution of the analyzed dataset, a second derivative of NIR spectra was calculated (Figure 1) and spectral regions with water contributions were omitted. The iPLS settings were screened in a similar way as for the Raman spectra, including 10–15 intervals with widths ranging from 15 to 50 data points. Finally, ten intervals consisting of 20 data points, each corresponding to the 9 cm^−1^ range of the NIR spectra, were selected for modeling (Tables S3 and S4 in Supplementary Material).

#### 2.2.2. Construction of Hybrid Models

As a part of a routine disease diagnosis, selected biochemical parameters of the analyzed sera were determined (Table 3). Among these variables are ESR, the levels of RBC, RF, MMP-3, TIMP-1, MPIF-1, HCC-4, and the concentration of Ca^2+^ ions. ESR, a known, nonspecific marker of acute phase response to inflammation, has long been used among diagnostic criteria of RA and disease activity monitoring [28]. It is considered important in the assessment of PsA progression [29]. Red blood cells have lately been involved in RA pathobiology because there is a positive correlation between RBC counts and joint pathology and with inflammatory biomarkers of the disease [30]. RF is an antibody, usually IgM, directed against the Fc portion of human IgG. High levels of RF in the blood are most often related to autoimmune diseases; however, it may also be detected in some healthy subjects. In some cases, patients suffering from autoimmune diseases may also have normal levels of rheumatoid factor [31]. Our previous study showed that a positive RF occurs in 72% of patients with RA, but in the case of PsA, the positive RF was observed in 7% of cases only [32]. All of this means that these parameters’ effectiveness in differentiating RA from PsA is not satisfactory for clinicians and there is a need to propose new ones that can be used as markers characterizing a given disease entity with higher sensitivity and specificity. In the case of diseases with an inflammatory background, such as RA and PsA, this seems not to be an easy task. Increasing attempts are also being made to select a panel of several diagnostic markers that would be used in the diagnosis of both diseases, which constitute the basis for constructing an appropriate diagnostic algorithm, allowing for a more specific differentiation of diseases with similar symptoms or etiology.

As can be expected, such a set of parameters may prove to be a better diagnostic tool than each analyzed parameter alone. Because RA and PsA are autoimmune diseases with an inflammatory background, we aimed to check whether any known biochemical parameter whose variability is observed in inflammatory diseases could be used in differential diagnostics of RA and PsA, supporting currently used diagnostic methods. In the present study, only eight parameters from the 19 previously selected [10] were finally used in the construction of models. Apart from the above-mentioned ESR, RBC, RF, MMP-3, and its tissue inhibitor, TIMP-1, were examined because they are present in the cleavage and remodeling of extracellular matrix and basal membrane components and in bone and cartilage destruction and connective tissue remodeling [33]; however, our previous study showed that the MMP-3/TIMP-1 ratios do not differ between the studied groups [10]. The other parameters analyzed were the chemokines MPIF-1 and HCC-4, which are small proteins involved in a variety of cells’ chemoattraction to the site of inflammation; therefore, they are important in the pathogenesis of RA [34,35]. Calcium ion concentration, the last parameter included in the construction of combined models, plays a crucial role in inflammation and inflammatory diseases [36]. It has been suggested that Ca^2+^ can regulate the metabolism of arachidonic acid in T cells and mediate synovial inflammation in RA patients during the inflammatory response [37]. It was also documented that Ca^2+^ mediates the infiltration of many immune cells during the development of RA, leading to an uncontrolled inflammatory response [38]. In our previous studies, we have demonstrated that combining some of these parameters with spectral data resulted in a better overall accuracy in differentiating RA, PsA, and control samples, with the OA parameter reaching 90%, whereas the best models based solely on the ATR spectra were characterized by an OA value of 82% [10].

To partially limit overrepresentation of spectral data compared to the number of biochemical variables included in hybrid modeling, only those spectral features derived from iPLS selection for which VIP exceeded a value of 1 were retained. This reduced the number of variables from 225 and 200 to 106 and 71 for Raman and NIR data, respectively.

#### 2.2.3. PLS-DA Modeling

**Figure 3:**
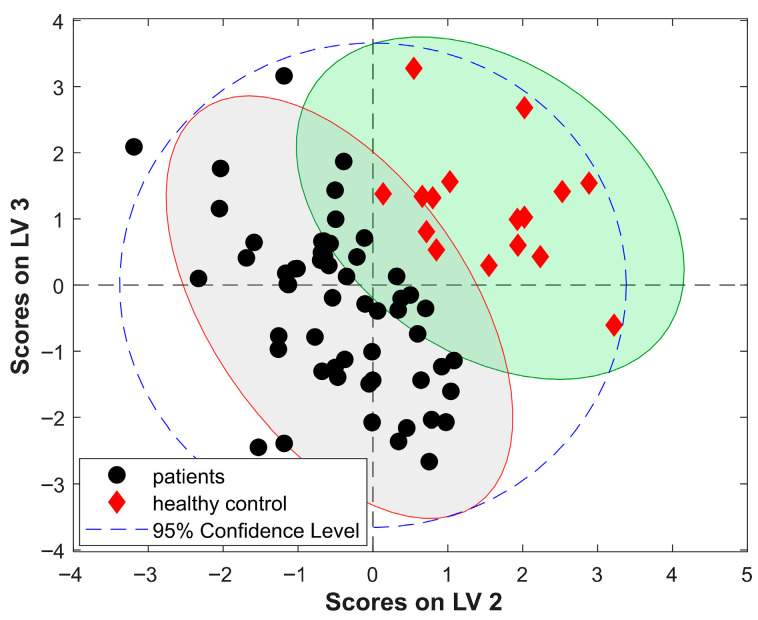
Scores plot for two-class PLS-DA model based on Raman spectra of serum lyophilizates; shaded areas correspond to the 95% confidence interval for patient samples (gray) and control group (green).

Using Raman and NIR spectra of serum lyophilizates, two-class discriminant models were constructed first, taking into account healthy individuals and all arthritis patients. The obtained sample distribution in the PLS-DA scores plots clearly shows that the information contained in the spectra of the studied material allows for the identification of samples originated from healthy subjects and those derived from patients with rheumatoid diseases of an inflammatory nature (Figure 3). These distributions also indicated the presence of several healthy control subjects who may show some symptoms of inflammation as well as objects assigned to arthritis patients with reduced disease activity, who could be potential outliers in the further three-class modeling. After we discarded evident outliers using a Hotteling T^2^ test, the remaining 60 objects were used for further PLS-DA modeling.

**Figure 4:**
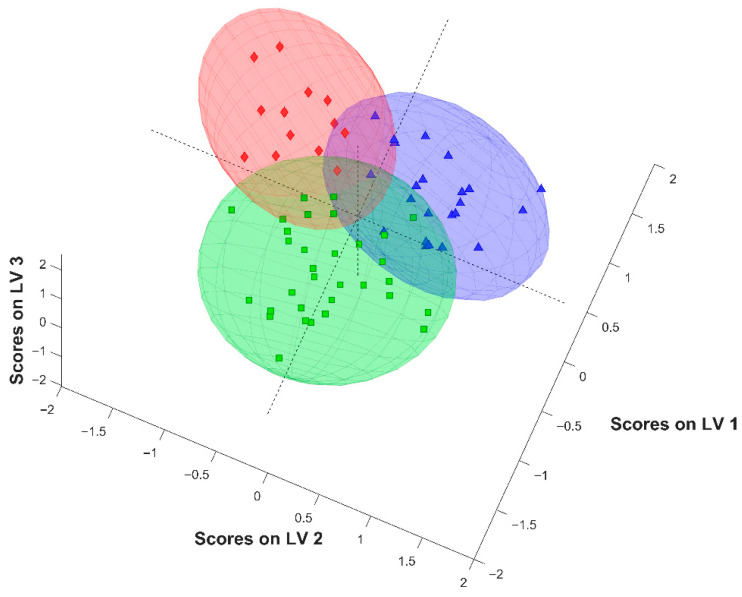
Scores plot for three-class PLS-DA model based on Raman spectra of serum lyophilizates using iPLS-selected variables; green—RA, blue—PsA, red—HC samples.

The development of three-class discriminant models is more demanding than that of two-class ones because features that differentiate healthy individuals from those suffering from inflammatory conditions do not distinguish RA patients from PsA patients, as both types of arthritis result in elevated values of inflammatory parameters of the blood. The score plot for the best PLS model constructed utilizing Raman data, based on the 225 spectral features selected by the iPLS method, is shown in Figure 4. A clear separation of three classes of objects was obtained, which indicates that the iPLS enables selection of a set of specific spectral variables allowing for separation of healthy subjects not only from patients subjected to inflammatory conditions but also individuals suffering from the two studied joint diseases. The selected variables cover ranges of Raman spectra of the sera characteristic for the ν_s_(CH) and amide III band vibration of proteins (Table S1 in Supplementary Material). The analysis conducted for a test set of spectra, not included in the construction of the PLS-DA models, confirmed that the developed classifier enables separation of samples originating from subjects of different pathophysiological characteristics, with an overall accuracy of 87.5% (Table 4 and Table S2 in Supplementary Material).

Using the second derivative of NIR spectra, the PLS-DA modeling was performed using 200 features selected by the iPLS algorithm, involving ten intervals of 20 data points (Tables S3 and S4 in Supplementary Material). The obtained model was characterized by similar performance compared to that of the Raman-based one but with slightly better results of the cross-validation (Table 4). Although NIR measurements of lyophilizates require careful control of the moisture of the studied samples, it seems that this technique has a high potential for wider application as an effective analytical tool for the study of biological material because lyophilization allows for long-term storage of samples without losing their properties. It is also important to note that NIR spectroscopy is a low-cost technique, which makes it a widely available source of data that can be used in differentiating types of arthritis. Using combined datasets, including biochemical and selected spectral data, PLS-DA models were constructed utilizing seven and four LVs for Raman and NIR data, respectively, and their quality was comparable (Table 4). The developed hybrid models are more robust than those based only on spectral data; the OA for test set prediction increased to 93.8% from 87.5% for models based on spectral data alone. A significant improvement of the OA_CV_ value to the level of 88.6–90.9% from 72.7 to 79.1% is also observed. The obtained results are in good agreement with those found for modeling based on FTIR ATR spectra [10]; the detailed parameters for the obtained models are listed in Table S5 in Supplementary Material.

#### 2.2.4. CP-ANN Modeling

In addition to PLS-DA modeling, the counter-propagation artificial neural networks (CP-ANN) were applied to construct discrimination models using the same Raman and NIR datasets, as in the case of PLS-DA models, as inputs. Because neural networks require optimization of their architecture settings, a screening procedure was carried out for spectral and combined data, including type of neighborhood correction, minimum and maximum values of the correction factors, network size (i.e., the number of neurons in the *x*- and *y*- dimensions), and the number of learning epochs. As a result, establishing optimal network architecture requires testing thousands of networks with different parameter combinations. Therefore, the screening procedure is noticeably more time-consuming than PLS-DA model construction.

**Figure 5:**
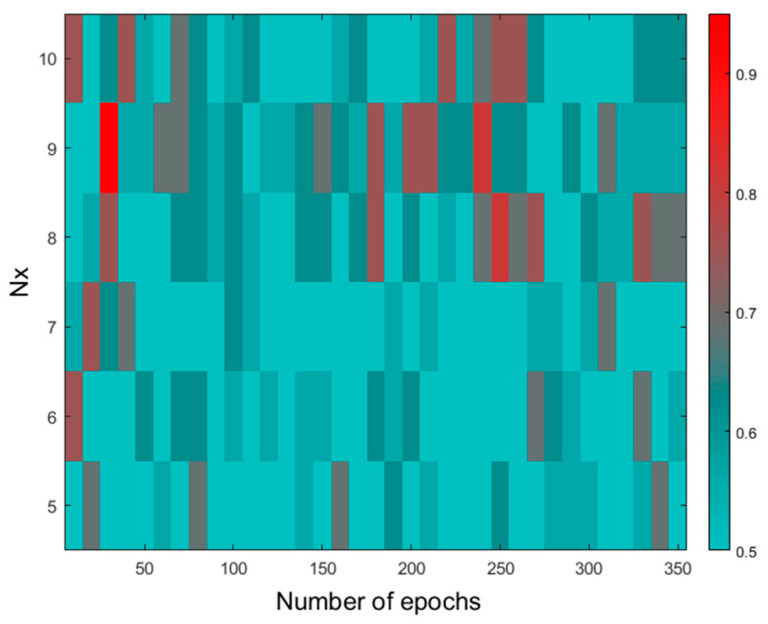
Screening of the CP-ANN architecture for Raman data—OA as a function of network dimension and number of learning epochs with maximum correction factor of 0.8.

The results of the CP-ANN parameter screening based on Raman data are shown in Figure 5. The best-performing network consisted of 81 neurons (9 × 9), trained using 30 epochs with the triangular type of neighborhood correction. This network was able to distinguish samples belonging to the three studied groups in a test set with an OA of 93.8% (Table 4 and Table S6 in Supplementary Material).

**Figure 6:**
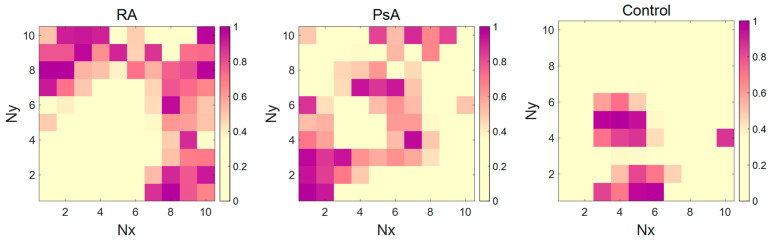
CP-ANN top maps for combined biochemical and Raman data—arrangement of neurons classifying patients with RA, PsA, and healthy subjects.

However, the number of other networks obtained for N_x_ and N_y_ varied from 8 to 10, and the number of epochs, ranging from 180 to 280, performed quite similarly. The best modeling performance for NIR data was achieved using an 81-neuroned network trained at 100 cycles. In comparison to Raman data modeling, slightly lower values of OA parameter were found for calibration and test sample prediction. For the combined datasets, CP-ANN modeling resulted in an overall accuracy for a test set equal to 87.5%, which means that elaborated CP-ANN models’ predictive ability is somewhat worse than that obtained applying PLS-DA modeling, but these models are characterized by a noticeably higher OA of cross-validation. The detailed parameters of the constructed classifiers are presented in Table S6 in the Supplementary Material. Top maps showing the arrangement of neurons classifying samples for the three studied groups based on the combined biochemical parameters and Raman data are presented in Figure 6, and those for other datasets are shown in Figures S3–S5 in the Supplementary Material.

## 3. Materials and Methods

### 3.1. Biological Material

The biological material was collected at the Department of Rheumatology and Internal Medicine of Wroclaw Medical University. In the course of study, sixty nine samples of freeze-dried blood sera collected from healthy volunteers (HC, *n* = 15) and patients with RA (*n* = 30) and PsA (*n* = 24), in whom the disease stage was assessed according to the European League Against Rheumatism (ACR/EULAR) and Classification Criteria for Psoriatic Arthritis (CASPAR) criteria, respectively, were used. The material was obtained from Caucasian donors with a median age of 48 (38–65), 42 (32–52), and 40 (37–51) years for RA, PsA, and control group, respectively; the interquartile range for Q_1_–Q_3_ is given in parentheses. Women comprised 90% of the RA group, 50% of PsA patients, and 83% of the healthy subjects. The median of the disease duration was 12 years for RA and 5 years for PsA patients. Comorbidities included Hashimoto’s thyroiditis (*n* = 4) and hypothyroidism (*n* = 4) in the RA group and hypothyroidism (*n* = 3) and hypertension (*n* = 3) in the PsA group. Available information regarding patient treatment indicates the use of two antirheumatic drugs: methotrexate and methylprednisolone. In the case of the former, treatment of varying durations was provided to 11 RA patients (37%) and 14 PsA patients (58%), while for the latter, the corresponding figures were 4 (13%) and 3 (13%), respectively. Detailed characteristics of the activity of arthritic diseases and determination of biochemical parameters of blood were described in Kokot et al. [10]. Among 27 parameters determined, including 19 serum cytokines/chemokines concentrations, 8 with the highest discriminatory capacity were selected (see Table 3) and used for the construction of hybrid models.

### 3.2. Spectroscopic Measurements

Vibrational spectra of the studied material were collected using an iS50 FTIR spectrometer (Thermo Nicolet, Madison, WI, USA). Raman data were obtained using FT Raman accessory (Thermo Nicolet, Madison, WI, USA), equipped with a CaF_2_ beam splitter and InGaAs detector. The lyophilizates placed in a conical holder (Figure S6 in Supplementary Material) were illuminated using an Nd:YVO_4_ laser, emitting a 1064 nm line with a power of 150 mW at the sample. Backscattered radiation was collected and spectra in the 100–3700 cm^−1^ range with a resolution of 8 cm^−1^ (3628 data points/spectrum) were obtained, accumulating 512 scans. NIR spectra in the 10,000–4000 cm^−1^ range were registered using a Collector (Thermo Scientific, Madison, WI, USA) diffuse-reflectance optical assembly mounted in the iS50 unit. A CaF_2_ beamsplitter and DTGS detector were used for the measurements. In total, 64 scans were accumulated and spectra with a resolution of 4 cm^−1^ were obtained, each consisting of 12,446 data points.

### 3.3. Computational Techniques

Partial least squares discriminant analysis (PLS-DA) is an efficient computational method used in solving classification problems by applying spectroscopic data [39]. It originated in the PLS technique, which is used to search for directions in the data projection that maximizes the covariance between variable matrix *X* and response matrix *Y*, which is achieved by decomposing the *X* and *Y* matrices

*X* = *TP*^*T*^ + *E*, (1)

*Y* = *UQ*^*T*^ + *F*, (2)

where *T* and *U* are the matrices of the new coordinates (scores), *P* and *Q* are loadings, and *E* and *F* are residuals representing the matrices’ unexplained variability. The decomposition of matrices, Equations (1) and (2), is carried out iteratively to maximize the covariance between matrices *T* and *U* [40]. Whereas in PLS modeling the parameters in the *Y* matrix can have continuous values, in PLS-DA, they are logical or integer values, defining class membership.

**Figure 7:**
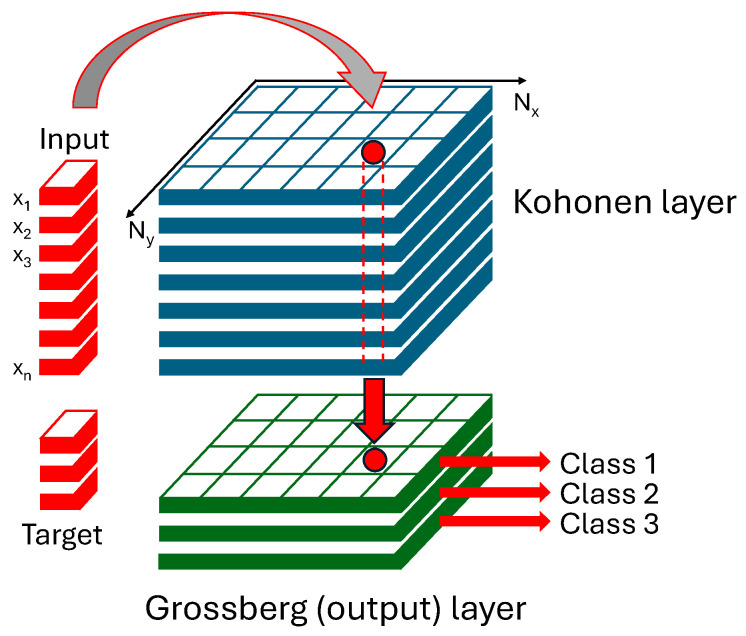
General scheme of CP-ANN network; Nx and Ny denote the number of neurons in the x and y directions, red arrows represent the class prediction process based on the assignment of input to a specific neuron.

CP-ANN belongs to the modeling techniques utilizing unsupervised and supervised learning to solve classification problems [41,42]. When the input vector (e.g., vibrational spectrum) is presented to the network, it is assigned to the specific neuron in the Kohonen layer with the set of predefined weights. This neuron is called a winning neuron. In the next step, the weights of this neuron and its neighbors are modified to provide a better match to the input vector. Each cycle of modifying the weights is called an epoch, and neurons containing similar data are therefore located near each other. After the network is stabilized, the Grossberg layer is activated by transferring the output from the Kohonen layer, resulting in a class membership representation. A general scheme of the counter-propagation network is shown in Figure 7.

To assess the applicability of the constructed PLS-DA and CP-ANN models (i.e., the obtained classifiers), a confusion matrix can be used. Based on the number of samples assigned to four classes, namely true positive (*TP*), false positive (*FP*), true negative (*TN*), and false negative (*FN*), a number of statistics characterizing the quality of the model can be computed. Among them, the most important are sensitivity and specificity. Sensitivity represents the probability with which a sample belonging to a given class will be correctly assigned to it, whereas specificity is the probability that a sample not belonging to a given class will not be incorrectly assigned to it. Other parameters that characterize obtained models are accuracy, which is the ratio of the number of correctly classified samples to all samples, and precision, which is the ratio of the number of true positives to the sum of true and false positives [43]. These metrics are calculated using the following equations:

*Precision* = *TP*/(*TP* + *FP*). (3)

*Sensitivity* (*recall*) = *TP*/(*TP* + *FN*). (4)

*Specificity* = *TN*/(*TN* + *FP*). (5)

*Accuracy* = (*TP* + *TN*)/(*TP* + *TN* + *FP* + *FN*). (6)

When constructing classification models that predict assignment to more than two groups of samples, statistics are calculated for each class. It is not uncommon for a given classifier to have significantly better metrics for one class than for another. To quickly assess the quality of multi-class models, the overall accuracy (OA), calculated as the sum of the products of the model’s sensitivity for a given class with its abundance divided by the total number of samples (n), can be used:

(7) $$OA\;=\;\frac{{Recall}_{Class1}n_{1}+{Recall}_{Class2}n_{2}+{Recall}_{Class3}n_{3}}{n}$$

### 3.4. Data Treatment

Spectral data were transferred to the MATLAB environment (ver. 24.2, R2024b. MathWorks, Natwick, MA, USA). Raman spectra were corrected using the standard normal variate (SNV) technique [44]. In the case of NIR data, the second derivative of spectra was computed using the Savitzky–Golay filter, applying 19 data points and a 3rd-order polynomial. PLS-DA models were constructed using the PLS-Toolbox (ver. 9.5, Eigenvector, Wenatchee, WA, USA). PLS-DA models were constructed utilizing whole spectral ranges of Raman and NIR spectra. Applying PLS-DA score plots in the Hotteling T^2^/Q residuals coordinate system, potential outliers were detected and removed from datasets [45]. Then samples were divided into calibration and validation sets using a Kennard–Stone algorithm [46], in which approximately 1/3 of the samples were selected for external validation. The iPLS algorithm working in a forward mode was used to select ranges of sera spectra displaying the highest discrimination potential [47]. To optimize the number and length of intervals, a screening procedure was performed for each type of spectral data. The number of latent variables used to build the PLS model was determined based on the plots of average classification errors. For hybrid modeling, autoscaled biochemical parameters and mean-centered spectra were further min–max scaled before merging to obtain data in the 0–1 range. To further reduce the number of variables, those with VIP > 1 [48] were included in the final modeling.

Neural networking simulations were performed using CP-ANN software developed by Zupan et al. [42]. Optimization of network architecture for each dataset involved screening of network size, i.e., the number of neurons in x- and y-dimensions in the 3–10 range, corresponding to network of 9–100 neurons, the number of learning epochs from 10 to 500, minimum and maximum values of correction factors in the 0.1–0.9 range, and various types of neighborhood correction.

To evaluate the performance of the obtained models, a leave-one-out cross-validation technique was used [49]. Parameters characterizing the quality of the obtained classifiers were calculated for calibration and validation sets based on Equations (3)–(7).

## 4. Conclusions

It was shown, for the first time, that Raman and NIR spectroscopies make discrimination of RA and PsA possible. The results obtained based on spectra of freeze-dried blood sera demonstrate an extraordinary potential of vibrational spectroscopy in RA and PsA differentiation. PLS-DA and CP-ANN models based on Raman and NIR spectra resulted in OA parameter values in the 81.3–93.8% range for the test set predictions. Combining spectroscopic data with values of selected biochemical parameters allowed for construction of even better hybrid models. The best PLS-DA and CP-ANN models differentiating samples originating from RA and PsA patients and the control group were characterized by an OA parameter for the test set in the 87.5–93.8% range. It should be noted that widely available NIR spectroscopy, under some circumstances, can be a real alternative to IR and Raman techniques frequently used in spectroscopy-supported medical diagnostics. It should be noted that the use of lyophilizates in this type of research is also not common. In this study it was shown that this form of biological material, which is standard for many medical analyses, can also be successfully used in spectroscopic diagnostics research.

Currently, due to no clear markers for rheumatoid or psoriatic arthritis, diagnosis is based on medical examination and history of disease. Overlapping symptoms between the two diseases provide another challenge in the clear distinction between them. The approach to the diagnostics presented in this study may complement methods of disease recognition. The presented models may be used as an additional tool by diagnosticians due to relatively low cost and short measurement time of vibrational spectra acquisition. Inclusion of selected biochemical parameters may also improve diagnostic capabilities of classifiers, as well as further reduce the time of analysis due to selection of only the most discriminant parameters. However, before clinical translation, further validation would be needed, involving larger study cohort.

## Figures and Tables

**Table 1:** Tentative band assignment in Raman spectrum of serum lyophilizates.

Position [cm^−1^]	Modes	Molecules	References
3300	ν(N-H), ν(O-H)		[11]
3060	aromatic ν(C-H)		[12]
2969	ν_as_(C-H)	methyl groups	[13]
2932	ν_as_(C-H)	methylene groups	[13]
2870	ν_s_(C-H)	methyl groups	[13]
2853	ν_s_(C-H)	methylene groups	[13]
1657	amide I band ν(C=O)	proteins	[14,15,16]
1606	ν(C=C)	tyrosine, phenylalanine	[15,16,17]
1585	ν(C=C)		[18]
1552	ν(C=C)	tryptophan	[16]
1448	δ(CH_2_)	phospholipids	[16]
1338		DNA purine bases	[14,15,19]
1270	amide III band ν(C-N)	proteins	[15,20]
1207	ν(C-C_6_H_5_)	tryptophan, phenylalanine	[15]
1176	δ(C-H)	tyrosine	[16]
1158	ν(C-N), ν(C-C)	proteins	[15,16,17]
1126	ν_s_ (C-N)	proteins	[15,17]
1105	ν(C-N)	proteins	[21]
1033	C−H in-plane	phenylalanine	[22]
1004	benzene ring breathing mode	phenylalanine	[14,16,19]
937	ν(C-C) in α-helix structure	proteins	[14,16,17]
894	ν(C-C) backbone		[14,15,16,17]
853	ring breathing mode	tyrosine	[19]
759	ring breathing mode	tryptophan	[15,17,19]
714		polysaccharides	[15,17]
644	C-C twisting	tyrosine	[14]
624	C-C twisting	phenylalanine	[15,17]

ν_s_—symmetric stretching vibration; ν_as_—asymmetric stretching vibration; δ—bending vibration.

**Table 2:** Tentative band assignment in NIR spectrum of serum lyophilizates.

Position [cm^−1^]	Position [nm]	Band Assignment	References
8399	1191	2nd overtone ν(C-H)	[23,24]
6648	1504	1st overtone ν(O-H), ν(N-H)	[24,25,26]
6331	1580	1st overtone ν(N-H)	[25]
5893	1697	1st overtone ν_as_(C-H)	[25]
5777	1731	1st overtone ν_s_(C-H)	[25,26]
5141	1945	combinatorial ν(O-H)	[23,24]
5063	1975	combinational ν(O-H)	[24]
4866	2055	combinational ν(N-H)	[26]
4610	2169	combinational ν(N-H)	[26]
4355	2296	combinational ν(C-H)	[26]
4261	2347	combinational ν(C-H)	[26,27]
4054	2467	combinational ν(C-H)	[27]

**Table 3:** Biochemical parameters included in the construction of hybrid models.

Parameter	Unit	Description	RA	PsA	Control
ESR	mm/h	Erythrocyte sedimentation rate	2.0–87.0	2.0–64.0	2.0–24.0
RBC	mln/mm^3^	Red blood cells	3.7–5.0	3.9–5.4	4.0–5.6
Ca^2+^	mg/dL	Calcium ions	6.9–10.2	8.9–10.4	8.8–9.9
MMP-3	ng/mL	matrix metalloproteinase-3	5.0–64.9	7.1–48.9	5.6–19.0
TIMP-1	pg/mL	Tissue inhibitor of metalloproteinase	141.0–340.2	127.8–275.7	65.3–175.1
RF	IU/mL	Rheumatoid factor	1.0–413.2	1.5–194.6	0.7–54.5
MPIF-1	pg/mL	Myeloid progenitor inhibitory factor 1	2.0–260.3	2.0–219.2	2.0–113.8
HCC-4	pg/mL	Liver-expressed chemokine	80.8–2204.1	174.3–1236.9	407.5–986.8

**Table 4:** Overall accuracy (OA) for PLS-DA and CP-ANN models.

Method	Parameter	Data Type (Number of Variables)			
		Raman Spectra	NIR Spectra	Biochemical Parameters + Raman Spectra	Biochemical Parameters + NIR Spectra
		(225)	(200)	(106)	(71)
	LVs	6	8	7	4
	OA_CAL_ (%)	100	100	100	100
PLS-DA	OA_CV_ (%)	72.7	79.1	90.9	88.6
	OA_TEST_ (%)	87.5	87.5	93.8	93.8
	architecture	TRI/0.8/9 × 9/30 *	TRI/0.8/9 × 9/100	TRI/0.5/10 × 10/140	TRI/0.3/8 × 8/270
	OA_CAL_ (%)	93.2	90.7	97.7	95.4
CP-ANN	OA_CV_ (%)	93.2	90.7	97.7	95.4
	OA_TEST_ (%)	93.8	81.3	87.5	87.5

* TRI—triangular type neighborhood correction/max correction/network size/number of epochs; OACAL—overall accuracy for training set, OATEST—overall accuracy for testing samples, OACV—overall accuracy for cross-validation.

## Data Availability

Datasets are available from the authors upon a reasonable request.

## Supplementary Materials

The following supporting information can be downloaded at: https://www.mdpi.com/article/10.3390/molecules30234513/s1, Figure S1: Raman spectra of blood serum lyophilizates and difference spectra; Figure S2: NIR spectra of blood serum lyophilizates and difference spectra; Figure S3: Top maps for CP-ANN model based on iPLS selected Raman data; Figure S4: Top maps for CP-ANN model based on iPLS selected NIR data; Figure S5: CP-ANN top maps for combined biochemical and NIR data; Figure S6: Blood serum lyophilizate placed in a conical holder; Figure S7: Hotelling T2 vs. Q residuals for PLS-DA scores for Raman data; Figure S8: ROC curves for PLS-DA models based on Raman data; Figure S9: ROC curves for PLS-DA models based on NIR data; Figure S10: ROC curves for PLS-DA models based on combined biochemical and Raman datasets; Figure S11: ROC curves for PLS-DA models based on combined biochemical and Raman datasets; Figure S12: Confusion matrices for PLS-DA models; Figure S13: Confusion matrices for CP-ANN models; Figure S14: Confusion matrices for CP-ANN models resulting from samples randomization (5 runs). Table S1: Screening of iPLS settings—selected spectral ranges of Raman spectra; Table S2: Screening of iPLS settings for PLS-DA modeling on the basis of Raman spectra; Table S3: Screening of iPLS settings—selected spectral ranges of NIR spectra; Table S4: Screening of iPLS settings for PLS-DA modeling on the basis of NIR spectra; Table S5: Parameters of PLS-DA models for combined datasets; Table S6: Parameters of CP-ANN models.

## Abbreviations

The following abbreviations are used in this manuscript:

ACR-EULAR	American College of Rheumatology and European League Against Rheumatism
ATR	Attenuated Total Reflectance
CASPAR	Classification Criteria for Psoriatic Arthritis
CP-ANN	Counter-Propagation Artificial Neural Network
CRP	C-reactive protein
CV	Cross-validation
DA	Discriminant Analysis
HC	Healthy control
iPLS	interval Partial Least Squares
IR	Infrared
LV	Latent Variable
LDA	Linear Discriminant Analysis
OA	Overall Accuracy
PLS	Partial Least Squares
PsA	Psoriatic arthritis
RA	Rheumatoid arthritis
RF	Rheumatoid Factor
VIP	Variability Importance in Projection
