# Supplementary material for: Discrimination of Rheumatoid and Psoriatic Arthritis Based on Raman and NIR Spectra Using Machine-Learning Algorithms
Source: Molecules. 2025 Nov 22;30(23):4513. doi: 10.3390/molecules30234513 (PMC12693699; doi:10.3390/molecules30234513)
Supplement: Supplementary file 1 [file molecules-30-04513-s001.zip › molecules-3925981-supplementary.pdf]

Discrimination of rheumatoid and psoriatic arthritis based on Raman and NIR spectra using machine-learning algorithms

Przemysław Cuprych [1], Izabela Kokot [2], Roman Szostak [1,*], Ewa Maria Kratz [2,*] and Sylwester Mazurek [1,*]

1 Department of Chemistry, University of Wroclaw, F. Joliot-Curie 14, 50-383 Wroclaw, Poland; przemyslaw.cuprych@uwr.edu.pl, roman.szostak@uwr.edu.pl, sylwester.mazurek@uwr.edu.pl

2 Department of Laboratory Diagnostics, Division of Laboratory Diagnostics, Faculty of Pharmacy, Wroclaw Medical University, Borowska Street 211A, 50-556 Wroclaw, Poland; izabela.kokot@ umw.edu.pl, ewa.kratz@umw.edu.pl

* Correspondence: roman.szostak@uwr.edu.pl (RS), sylwester.mazurek@uwr.edu.pl (SM) and ewa.kratz@umw.edu.pl (EMK)

Supplementary materials

List of content:

Table S1. Screening of iPLS settings: selected spectral ranges of Raman spectra

Table S2. Screening of iPLS settings for PLS-DA modeling on the basis of Raman spectra

Table S3. Screening of iPLS settings: selected spectral ranges of NIR spectra

Table S4. Screening of iPLS settings for PLS-DA modeling on the basis of NIR spectra

Table S5. Parameters of PLS-DA models for combined data sets

Table S6. Parameters of CP-ANN models

Figure S1. Average Raman spectra of blood serum lyophilizates and difference spectra

Figure S2. Average NIR spectra of blood serum lyophilizates and difference spectra

Figure S3: Top maps for CP-ANN model based on iPLS selected Raman data

Figure S4: Top maps for CP-ANN model based on iPLS selected NIR data

Figure S5: CP-ANN top maps for combined biochemical and NIR data

Figure S6. Blood serum lyophilizate placed in a conical holder.

Figure S7. Hotelling T2 vs Q residuals for PLS-DA scores for Raman data.

Figure S8. ROC curves for PLS-DA models based on Raman data

Figure S9. ROC curves for PLS-DA models based on NIR data.

Figure S10. ROC curves for PLS-DA models based on combined biochemical and Raman datasets.

Figure S11. ROC curves for PLS-DA models based on combined biochemical and Raman datasets.

Figure S12. Confusion matrices for PLS-DA models.

Figure S13. Confusion matrices for CP-ANN models.

Figure S14. Confusion matrices for CP-ANN models resulting from samples randomization (5 runs)

Table S1. Screening of iPLS settings: selected spectral ranges of Raman spectra

Number of intervals	Width of intervals	Selected spectral ranges [cm^{-1}]
15	10	527-536, 633-642, 778-787, 1270-1279, 1569-1577, 1646-1655, 1742-1751, 2253-2272, 2350-2358, 2437-2445, 2620-2628, 2822-2831, 2919-2927, 3149-3158
10	15	778-806, 2210-2223, 2297-2310, 2514-2527, 2615-2628, 3005-3019, 3107-3135, 3179-3193
15	15	1125-1139, 1255-1269, 1313-1327, 1342-1356, 1979-1992, 2051-2064, 2181-2195, 2572-2585, 2615-2643, 2875-2889, 3136-3164, 3425-3453
20	15	662-676, 778-806, 879-893, 1140-1153, 1255-1269, 1588-1616, 1675-1688, 1819-1833, 2210-2238, 2297-2310, 2412-2426, 2514-2527 2615-2628 3005-3019 3107-3135, 3179-3193
15	20	508-526, 605-623, 682-700, 817-835, 894-912, 1125-1144, 1183-1201, 1241-1259, 1877-1896, 2514-2532, 2572-2590, 2610-2628 2803-2821, 2996-3014, 3111-3130

Table S2. Screening of iPLS settings for PLS-DA modeling on the basis of Raman spectra

		Class					
Number of intervals			15	10	**15**	20	15
Width of intervals			10	15	**15**	15	20
LVs			3	4	**6**	7	2
Calibration	Sensitivity [%]	HC	87.5	100	**100**	100	62.5
		RA	90.0	80.0	**100**	100	90.0
		PsA	93.8	100	**100**	100	68.8
	Specificity [%]	HC	97.2	94.4	**100**	100	97.2
		RA	91.7	100	**100**	100	79.2
		PsA	96.4	92.9	**100**	100	85.7
	Accuracy [%]	HC	95.5	95.5	**100**	100	90.9
		RA	90.9	90.9	**100**	100	84.1
		PsA	95.5	95.5	**100**	100	79.5
	F1 score	HC	87.5	88.9	**100**	100	71.4
		RA	90.0	88.9	**100**	100	83.7
		PsA	93.8	94.1	**100**	100	71.0
	OA [%]		90.5	91.9	**100**	100	75.6
CV	Sensitivity [%]	HC	50.0	37.5	**75.0**	62.5	25.0
		RA	70.0	55.0	**75.0**	90.0	60.0
		PsA	62.5	43.8	**68.8**	50.0	50.0
	Specificity [%]	HC	91.7	80.6	**77.8**	91.7	91.7
		RA	66.7	54.2	**83.3**	58.3	58.3
		PsA	82.1	82.1	**100**	100	67.9
	Accuracy [%]	HC	84.1	72.7	**77.3**	86.4	79.5
		RA	68.2	54.5	**79.5**	72.7	59.1
		PsA	75.0	68.2	**88.6**	81.8	61.4
	F1 score	HC	53.3	33.3	**54.5**	62.5	30.8
		RA	66.7	52.4	**76.9**	75.0	57.1
		PsA	64.5	50.0	**81.5**	66.7	48.5
	OA [%]		62.0	46.6	**72.7**	69.8	47.0
Test	Sensitivity [%]	HC	100	25.0	**75.0**	50.0	50.0
		RA	66.7	66.7	**100**	83.3	83.3
		PsA	66.7	83.3	**83.3**	66.7	50.0
	Specificity [%]	HC	91.7	75.0	**91.7**	83.3	91.7
		RA	80.0	80.0	**100**	80.0	70.0
		PsA	90.0	90.0	**90.0**	90.0	80.0
	Accuracy	HC	93.8	62.5	**87.5**	75.0	81.3
		RA	75.0	75.0	**100**	81.3	75.0
		PsA	81.3	87.5	**87.5**	81.3	68.8
	F1 score	HC	88.9	25.0	**75.0**	50.0	57.1
		RA	66.7	66.7	**100**	76.9	71.4
		PsA	72.7	83.3	**83.3**	72.7	54.5
	OA [%]		76.0	60.2	**87.5**	68.8	63.5

Table S3. Screening of iPLS settings: selected spectral ranges of NIR spectra

Number of intervals	Width of intervals	Selected spectral ranges [cm^{-1}]
10	30	4521-4535, 4796-4810, 4882-4896, 5765-5779, 6213-6227, 6285-6299, 6358-6386, 7544-7558, 7746-7760
10	15	4369-4376, 4608-4614, 4788-4795, 5078-5084, 5172-5178, 5989-5996, 6379-6386, 6965-6972, 7428-7435, 8137-8143
10	20	4299-4308, 4685-4694, 5080-5089, 5581-5591, 5601-5610, 6054-6063, 6536-6545, 7057-7066, 7124-7133, 7298-7307
15	15	4311-4318, 4369-4376, 4608-4614, 4788-4795, 5078-5084, 5172-5178, 5729-5735, 5989-5996, 6379-6386, 6538-6545, 6835-6842, 6965-6972, 7312-7319, 7428-7435, 8137-8143
15	20	4656-4665, 4694-4704, 4791-4800, 5167-5176, 5398-5407, 5437-5446, 5678-5687, 5765-5783, 5996-6005, 6218-6237, 6362-6372, 6719-6728, 7953-7963
10	50	4675-4699, 4747-4771, 5157-5205, 5639-5687, 6845-6868, 6941-6965, 7399-7423, 7592-7616

Table S4. Screening of iPLS settings for PLS-DA modeling on the basis of NIR spectra

		Class						
Number of intervals			10	10	**10**	15	15	10
Width of intervals			30	15	**20**	15	20	50
LVs			6	5	**8**	7	5	6
Calibration	Sensitivity [%]	HC	100	100	**100**	100	85.7	100
		RA	100	90.5	**100**	100	100	100
		PsA	100	100	**100**	100	100	100
	Specificity [%]	HC	100	97.2	**100**	100	100	100
		RA	100	100	**100**	100	95.5	100
		PsA	100	96.4	**100**	100	100	100
	Accuracy [%]	HC	100	97.7	**100**	100	97.7	100
		RA	100	95.3	**100**	100	97.7	100
		PsA	100	97.7	**100**	100	100	100
	F1 score	HC	100	93.3	**100**	100	92.3	100
		RA	100	95.0	**100**	100	97.7	100
		PsA	100	96.8	**100**	100	100	100
	OA [%]		100	96.1	**100**	100	96.0	100
CV	Sensitivity [%]	HC	42.9	71.4	**71.4**	100	71.4	57.1
		RA	85.7	66.7	**81.0**	90.5	81.0	90.5
		PsA	80.0	66.7	**80.0**	80.0	60.0	73.3
	Specificity [%]	HC	97.2	91.7	**94.4**	94.4	86.1	94.4
		RA	77.3	72.7	**86.4**	90.9	81.8	77.3
		PsA	85.7	82.1	**85.7**	96.4	89.3	92.9
	Accuracy [%]	HC	88.4	88.4	**90.7**	95.3	83.7	88.4
		RA	81.4	69.8	**83.7**	90.7	81.4	83.7
		PsA	83.7	76.7	**83.7**	90.7	79.1	86.0
	F1 score	HC	54.5	66.7	**71.4**	87.5	58.8	61.5
		RA	81.8	68.3	**82.9**	90.5	81.0	84.4
		PsA	77.4	66.7	**77.4**	85.7	66.7	78.6
	OA [%]		71.9	68.0	**79.1**	89.9	71.7	75.7
Test	Sensitivity [%]	HC	25.0	0.0	**75.0**	0.0	0.0	50.0
		RA	50.0	66.7	**100**	83.3	50.0	33.3
		PsA	50.0	33.3	**83.3**	33.3	66.7	33.3
	Specificity [%]	HC	75.0	66.7	**100**	75.0	91.7	58.3
		RA	70.0	70.0	**80,0**	70,0	50.0	80.0
		PsA	70.0	70.0	**100**	70.0	70.0	70.0
	Accuracy [%]	HC	62.5	50.0	**93.8**	56.3	68.8	56.3
		RA	62.5	68.8	**87.5**	75.0	50.0	62.5
		PsA	62.5	56.3	**93.8**	56.3	68.8	56.3
	F1 score	HC	0.25	-	**85.7**	-	-	36.4
		RA	0.50	61.5	**85.7**	71.4	42.9	40.0
		PsA	0.50	36.4	**90.9**	36.3	61.5	36.4
	OA [%]		43.0	37.5	**87.5**	44.3	41.1	38.0

Table S5. Parameters of PLS-DA models for combined data sets

		Class	Biochemical parameters + Raman spectra	Biochemical parameters + NIR spectra
Number of intervals			15	10
Width of intervals			15	20
LVs			7	4
Calibration	Sensitivity [%]	HC	100	100
		RA	100	100
		PsA	100	100
	Specificity [%]	HC	100	100
		RA	100	100
		PsA	100	100
	Accuracy [%]	HC	100	100
		RA	100	100
		PsA	100	100
	F1 score	HC	100	100
		RA	100	100
		PsA	100	100
	OA [%]		**100**	**100**
CV	Sensitivity [%]	HC	87.5	90.0
		RA	95.0	95.0
		PsA	87.5	78.6
	Specificity [%]	HC	97.2	97.1
		RA	87.5	91.7
		PsA	100	93.3
	Accuracy [%]	HC	95.5	95.5
		RA	90.9	93.2
		PsA	95.5	88.6
	F1 score	HC	87.5	90.0
		RA	90.5	92.7
		PsA	93.3	81.5
	OA [%]		**90.9**	**88.5**
Test	Sensitivity [%]	HC	75.0	100
		RA	100	83.3
		PsA	100	100
	Specificity [%]	HC	100	91.7
		RA	100	100
		PsA	90.0	100
	Accuracy [%]	HC	93.8	93.8
		RA	100	93.8
		PsA	93.8	100
	F1 score	HC	85.7	88.9
		RA	100	90.9

Table S6. Parameters of CP-ANN models

		Class	Raman spectra	NIR spectra	Biochemical parameters + Raman spectra	Biochemical parameters + NIR spectra
Network architecture			TRI/0.8/9×9/30	TRI/0.8/9×9/100	TRI/0.5/10×10/140	TRI/0.3/8×8/270
Calibration	Sensitivity [%]	HC	87.5	100	100	85.7
		RA	95.0	81.0	100	95.2
		PsA	94.0	100	94.0	100
	Specificity [%]	HC	100	94.4	97.2	100
		RA	91.7	100	100	95.5
		PsA	96.4	92.9	100	96.4
	Accuracy [%]	HC	97.7	95.3	97.7	97.7
		RA	93.2	90.7	100	95.3
		PsA	95.5	95.3	97.7	97.7
	F1 score	HC	93.3	87.5	94.1	92.3
		RA	92.7	89.5	100	95.2
		PsA	93.8	93.8	96.8	96.8
	**OA [%]**		**93.2**	**90.7**	**97.7**	**95.4**
CV	Sensitivity [%]	HC	87.5	100	100	85.7
		RA	95.0	85.7	100	95.2
		PsA	94.0	93.0	94.0	100
	Specificity [%]	HC	100	94.4	97.2	100
		RA	91.7	95.5	100	95.5
		PsA	96.4	96.4	100	96.4
	Accuracy [%]	HC	97.7	95.3	97.7	97.7
		RA	93.2	90.7	100	95.3
		PsA	95.5	95.3	97.7	97.7
	F1 score	HC	93.3	87.5	94.1	92.3
		RA	92.7	90.0	100	95.2
		PsA	93.8	93.3	96.8	96.8
	**OA [%]**		**93.2**	**90.7**	**97.7**	**95.4**
Test	Sensitivity [%]	HC	75.0	75.0	100	75.0
		RA	100	83.3	100	100
		PsA	100	83.0	67.0	83.0
	Specificity [%]	HC	100	100	91.7	91.7
		RA	100	80.0	90.0	90.0
		PsA	90	90.0	100	100
	Accuracy [%]	C	93.8	93.8	93.8	87.5
		RA	100	81.3	93.8	93.8
		PsA	93.8	87.5	87.5	93.8
	F1 score	HC	85.7	85.7	88.9	75.0
		RA	100	76.9	92.3	92.3
		PsA	92.3	83.3	80.0	90.9
	**OA [%]**		**93.8**	**81.3**	**87.5**	**87.5**

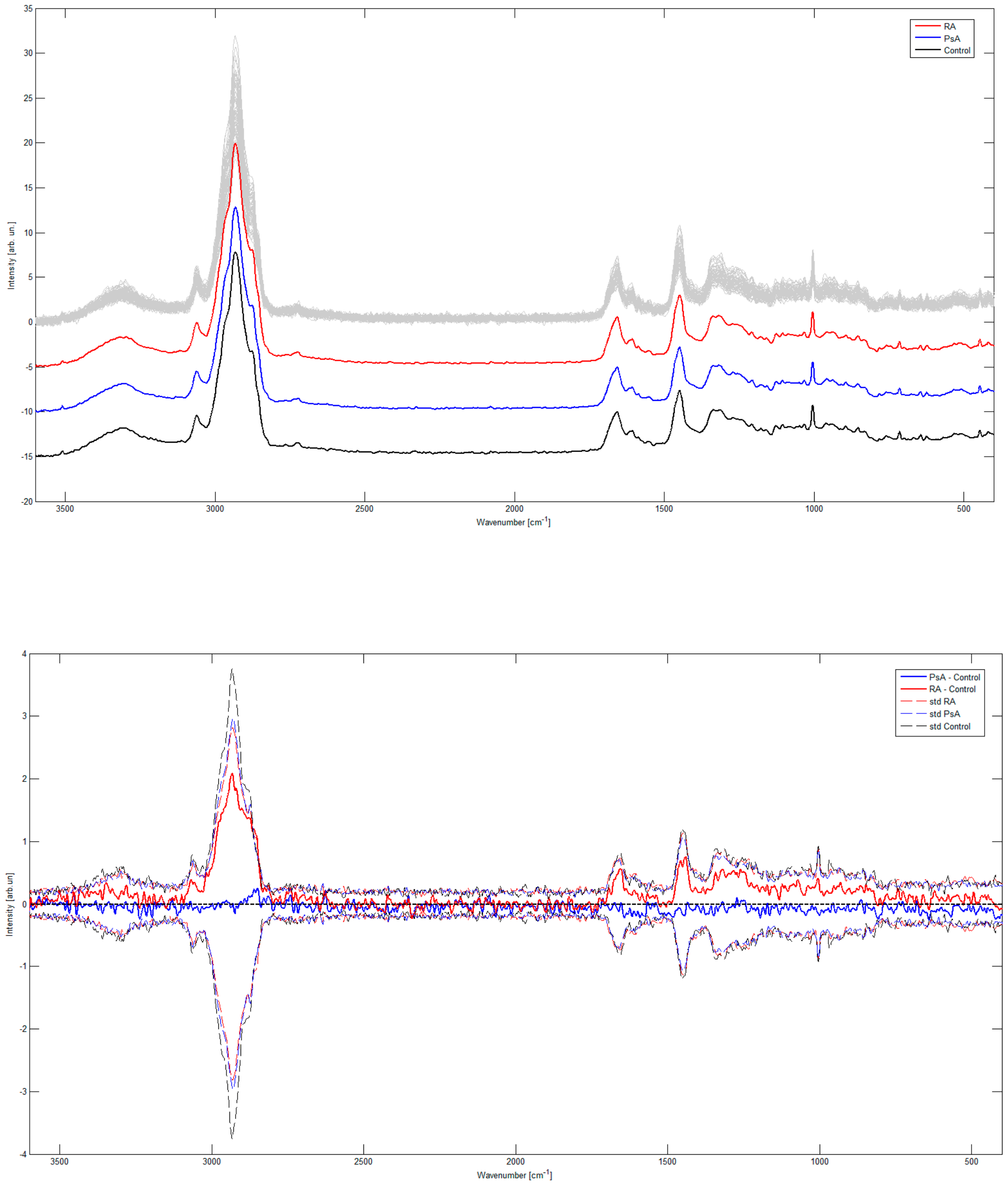

Figure S1. Average Raman spectra of blood serum lyophilizates (top) and difference spectra (bottom). Red – rheumatoid arthritis; blue – psoriatic arthritis; black – healthy controls.

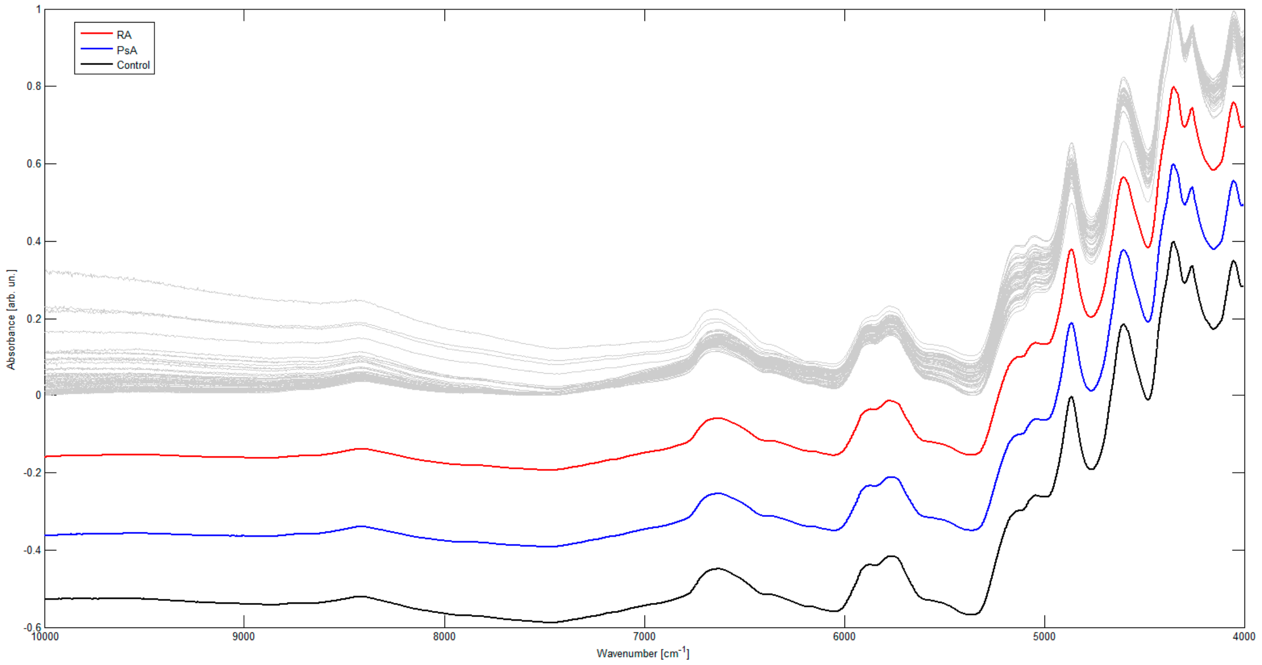

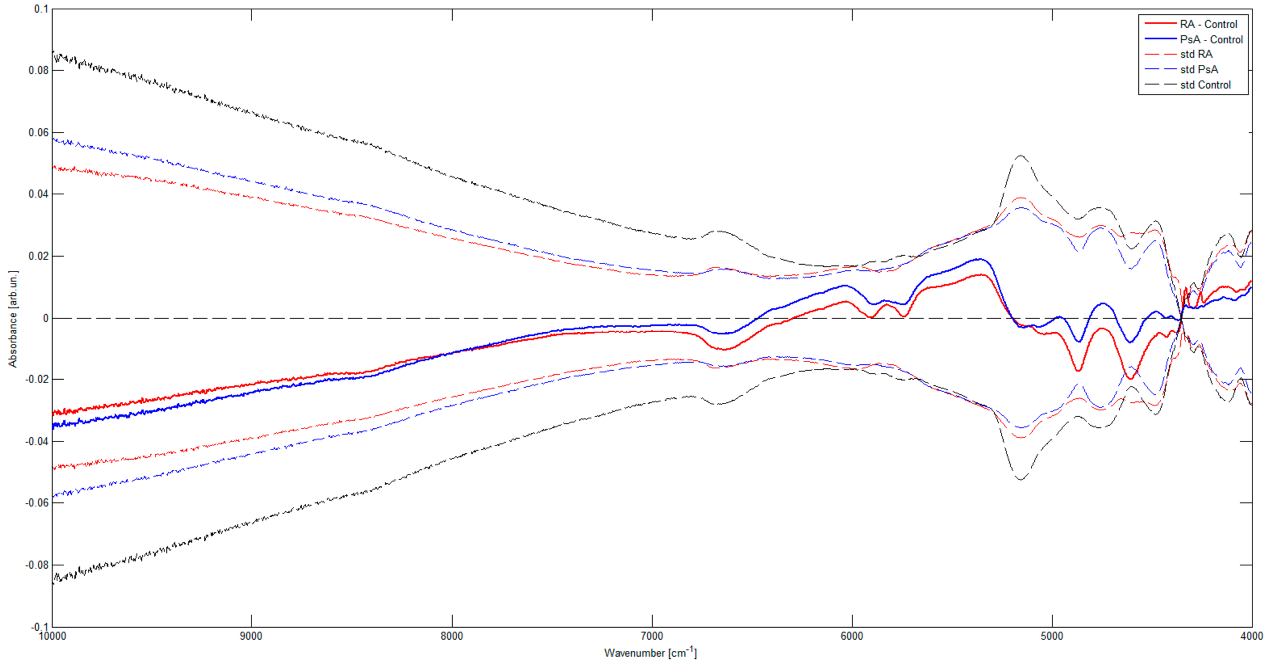

Figure S2. Average NIR spectra of blood serum lyophilizates (top) and difference spectra (bottom). Red – rheumatoid arthritis; blue – psoriatic arthritis; black – healthy controls.

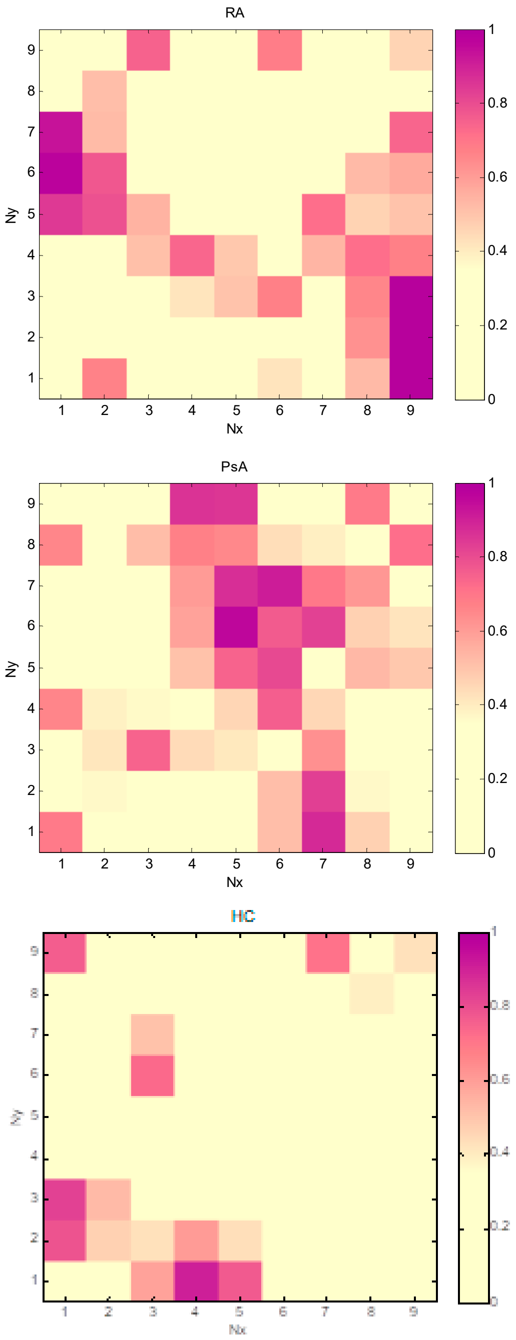

Figure S3. Top maps for CP-ANN model based on iPLS selected Raman data

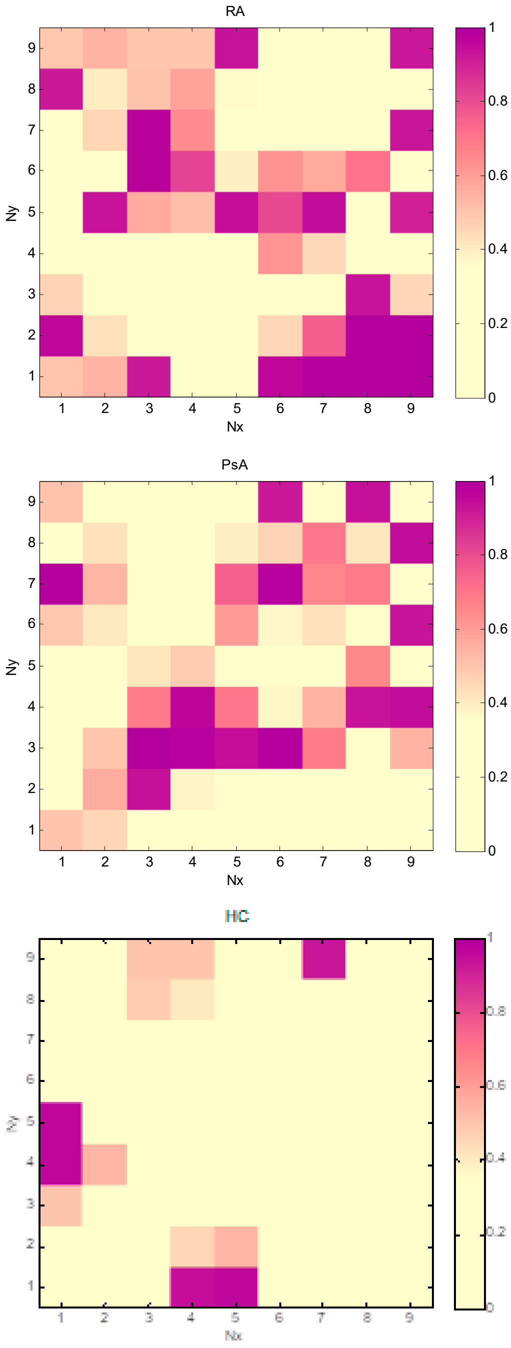

Figure S4. Top maps for CP-ANN model based on iPLS selected NIR data

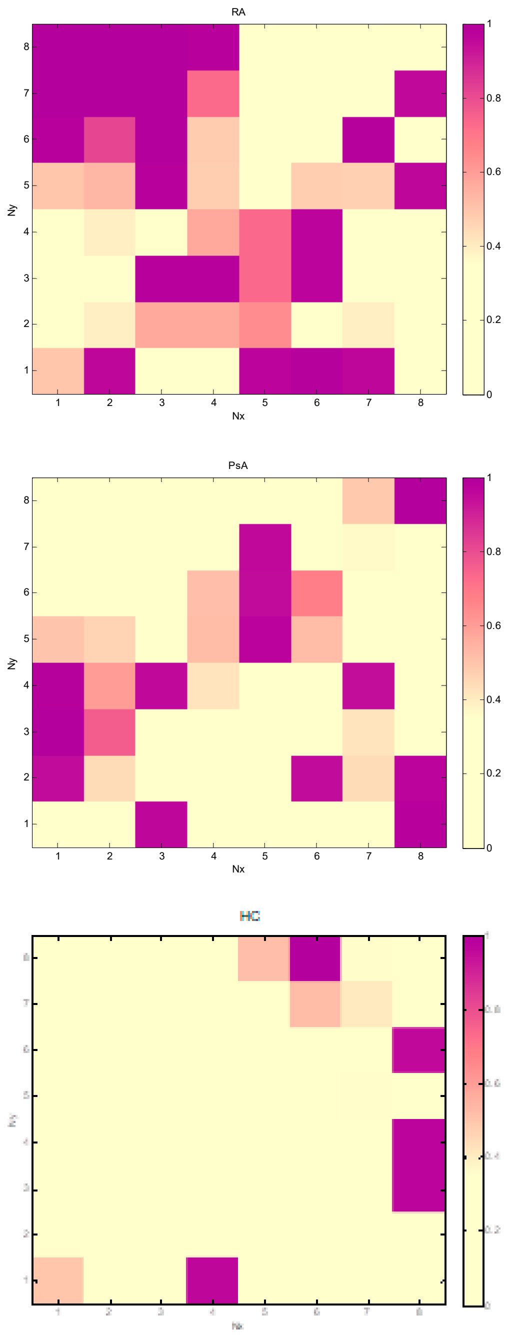

Figure S5. CP-ANN top maps for combined biochemical and NIR data

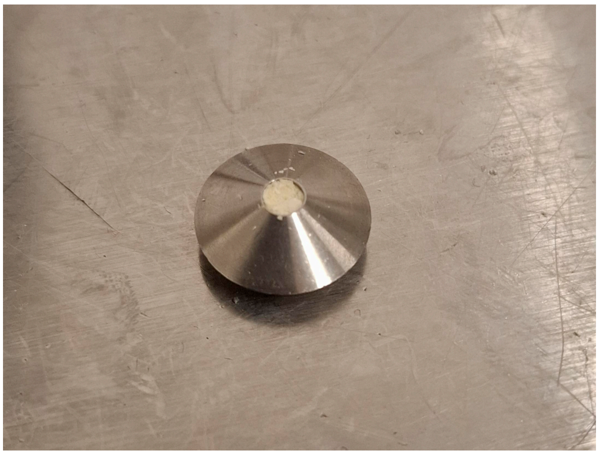

Figure S6. Blood serum lyophilizate placed in a conical holder.

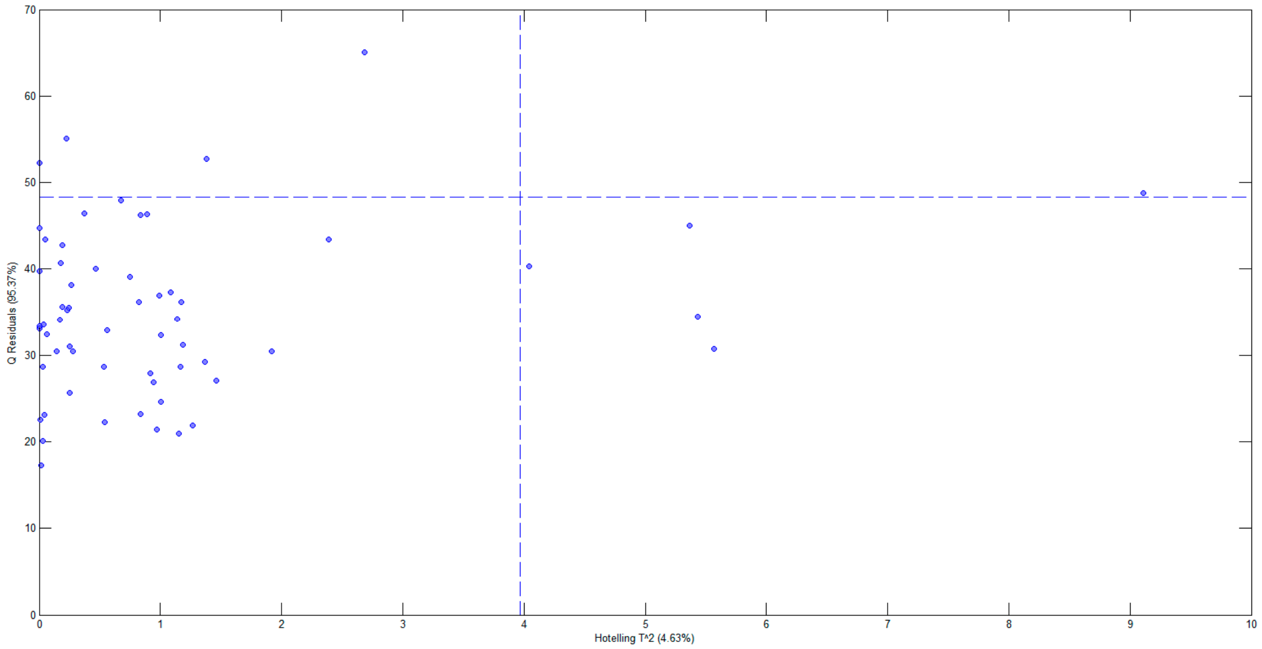

Figure S7. Hotelling T^2 vs Q residuals for PLS-DA scores for Raman data.

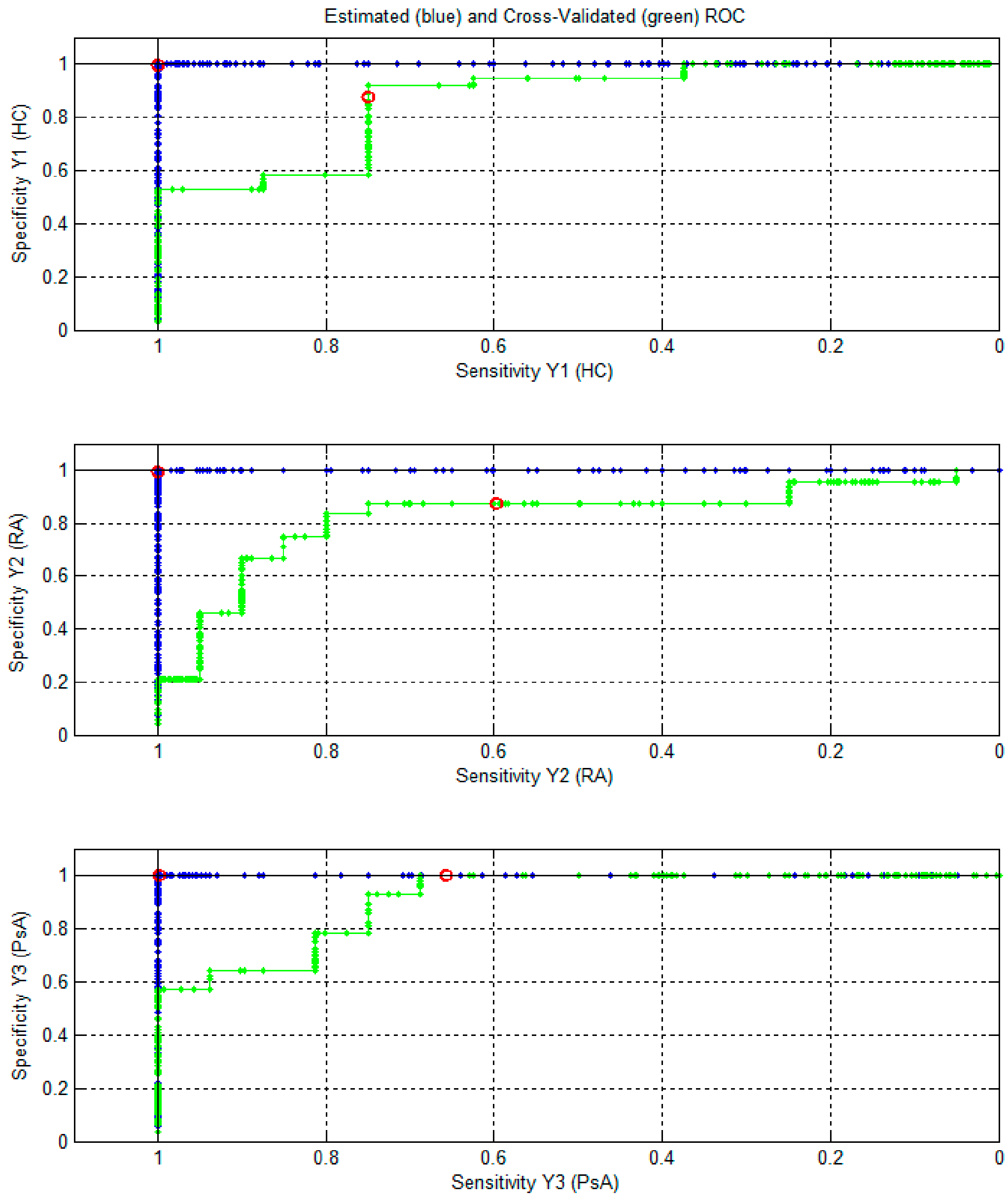

Figure S8. ROC curves for PLS-DA models based on Raman data.

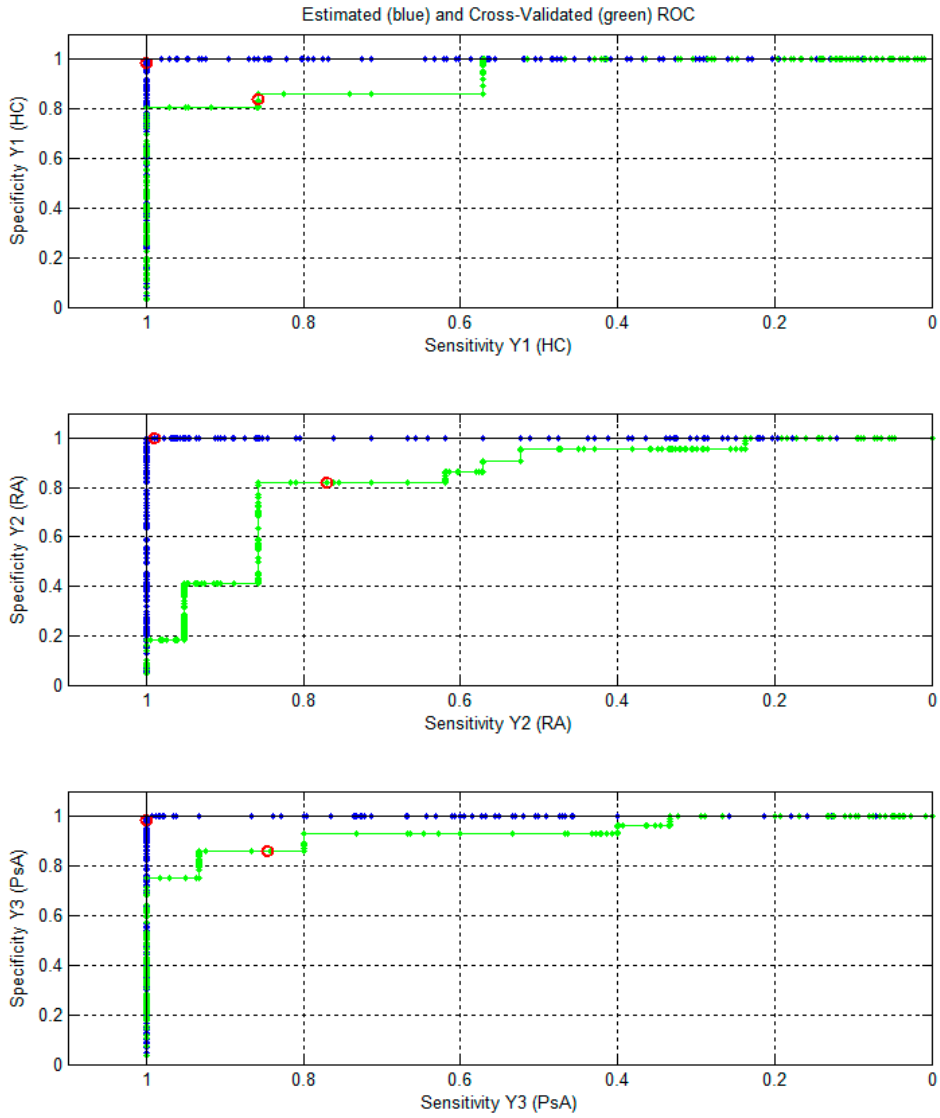

Figure S9. ROC curves for PLS-DA models based on NIR data.

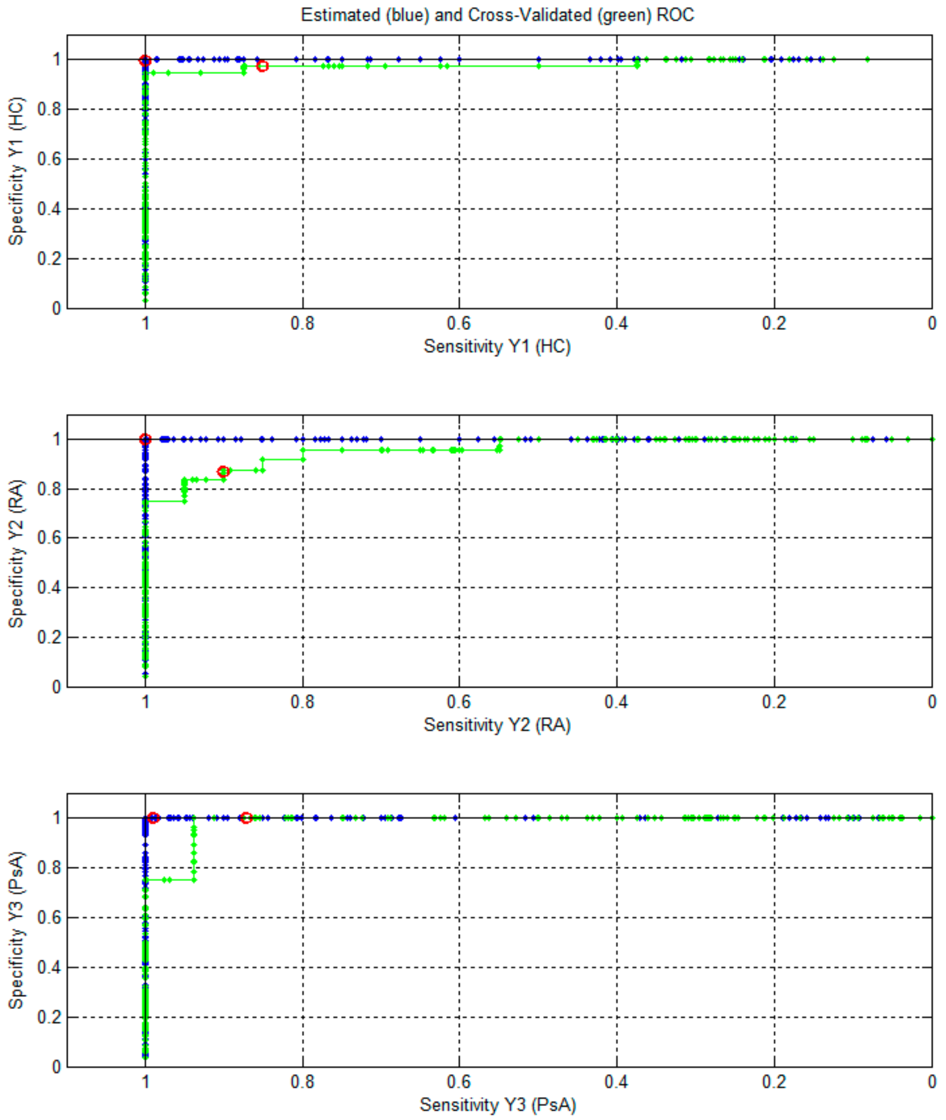

Figure S10. ROC curves for PLS-DA models based on combined biochemical and Raman datasets.

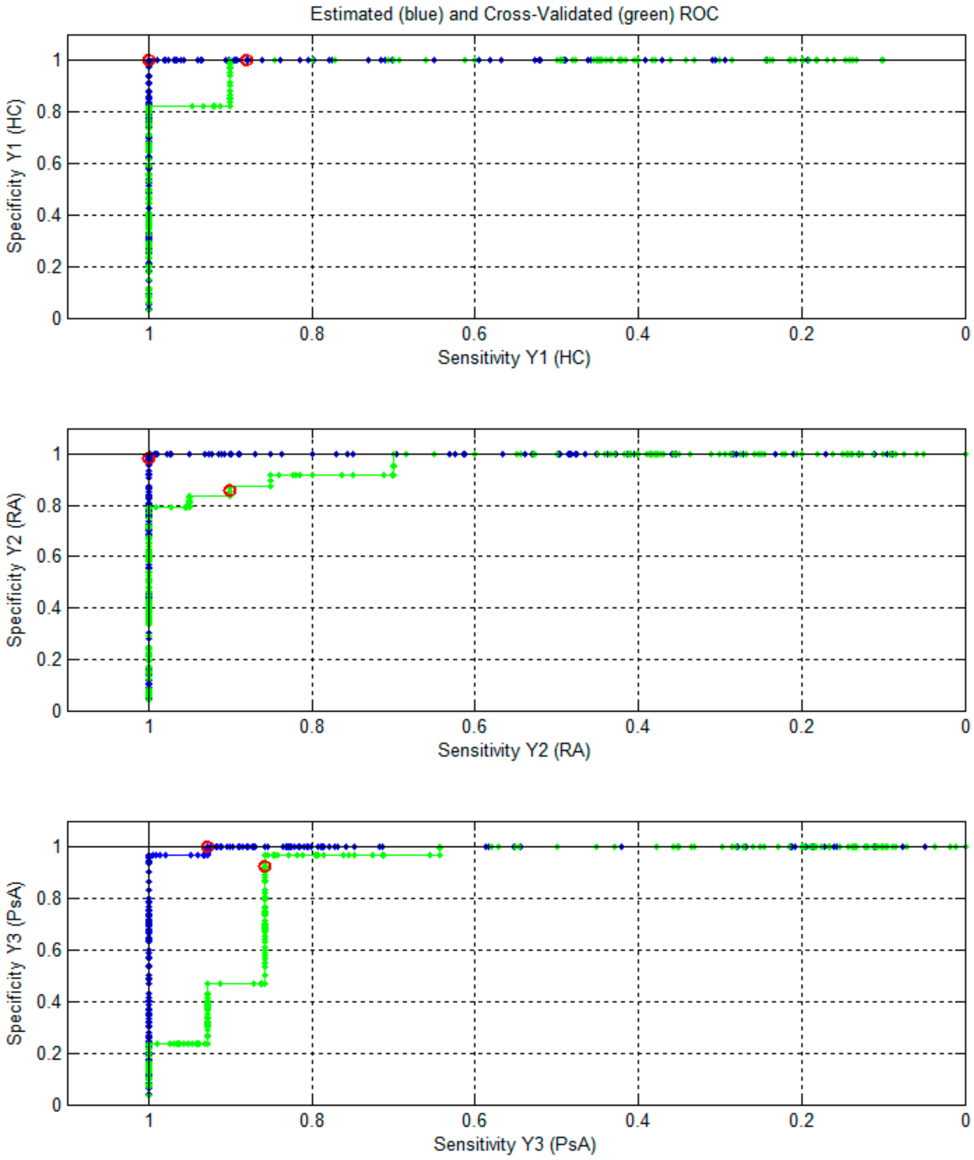

Figure S11. ROC curves for PLS-DA models based on combined biochemical and Raman datasets.

	Raman				NIR				Biological + Raman				Biological + NIR		

calibration

	HC	RA	PsA		HC	RA	PsA		HC	RA	PsA		HC	RA	PsA
HC	8	0	0	HC	7	0	0	HC	8	0	0	HC	10	0	0
RA	0	20	0	RA	0	21	0	RA	0	20	0	RA	0	20	0
PsA	0	0	16	PsA	0	0	15	PsA	0	0	16	PsA	0	0	14

CV

	HC	RA	PsA		HC	RA	PsA		HC	RA	PsA		HC	RA	PsA
HC	6	5	3	HC	5	1	1	HC	7	1	0	HC	9	0	1
RA	2	15	2	RA	1	17	2	RA	1	19	2	RA	0	19	2
PsA	0	0	11	PsA	1	3	12	PsA	0	0	14	PsA	1	1	11

test

	HC	RA	PsA		HC	RA	PsA		HC	RA	PsA		HC	RA	PsA
HC	3	0	1	HC	3	0	0	HC	3	0	0	HC	4	1	0
RA	0	6	0	RA	1	6	1	RA	0	6	0	RA	0	5	0
PsA	1	0	5	PsA	0	0	5	PsA	1	0	6	PsA	0	0	6

Figure S12. Confusion matrices for PLS-DA models.

	Raman				NIR				Biological + Raman				Biological + NIR		
**calibration**															
	RA	PsA	HC		RA	PsA	HC		RA	PsA	HC		RA	PsA	HC
RA	19	1	1	RA	17	0	0	RA	20	0	0	RA	20	0	1
PsA	1	15	0	PsA	2	15	0	PsA	0	15	0	PsA	1	15	0
HC	0	0	7	HC	2	0	7	HC	0	1	8	HC	0	0	6
**CV**															
	RA	PsA	HC		RA	PsA	HC		RA	PsA	HC		RA	PsA	HC
RA	19	1	1	RA	18	1	0	RA	20	0	0	RA	20	0	1
PsA	1	15	0	PsA	1	14	0	PsA	0	15	0	PsA	1	15	0
HC	0	0	7	HC	2	0	7	HC	0	1	8	HC	0	0	6
**test**															
	RA	PsA	HC		RA	PsA	HC		RA	PsA	HC		RA	PsA	HC
RA	6	0	0	RA	5	1	1	RA	6	1	0	RA	6	0	1
PsA	0	6	1	PsA	1	5	0	PsA	0	4	0	PsA	0	5	0
HC	0	0	3	HC	0	0	3	HC	0	1	4	HC	0	1	3

Figure S13. Confusion matrices for CP-ANN models.

Raman data

CV

	RA	PsA	HC		RA	PsA	HC		RA	PsA	HC		RA	PsA	HC		RA	PsA	HC		
RA	18	1	0	RA	19	1	1	RA	19	2	1	RA	20	1	0	RA	18	0	0		
PsA	1	15	0	PsA	1	15	1	PsA	1	13	0	PsA	0	15	1	PsA	0	16	1		
HC	1	0	8	HC	0	0	6	HC	0	1	7	HC	0	0	7	HC	2	0	7	Mean OA	std
	OA=	93,2			OA=	90,9			OA=	88,7			OA=	95,5			OA=	93,2		92,3	2,58747

test

	RA	PsA	HC		RA	PsA	HC		RA	PsA	HC		RA	PsA	HC		RA	PsA	HC		
RA	5	1	1	RA	5	3	0	RA	5	1	1	RA	5	0	0	RA	5	0	0		
PsA	1	4	0	PsA	1	3	3	PsA	1	5	0	PsA	0	5	1	PsA	0	6	1		
HC	0	1	3	HC	0	0	1	HC	0	0	3	HC	1	1	3	HC	1	0	3	Mean OA	std
	OA=	75,0			OA=	87,5			OA=	81,3			OA=	81,3			OA=	87,5		82,5	5,223217

NIR data

CV

	RA	PsA	HC		RA	PsA	HC		RA	PsA	HC		RA	PsA	HC		RA	PsA	HC		
RA	19	0	0	RA	20	0	0	RA	18	0	0	RA	19	0	1	RA	18	0	0		
PsA	2	15	0	PsA	1	15	0	PsA	3	15	1	PsA	2	15	0	PsA	1	15	0		
HC	0	0	7	HC	0	0	7	HC	0	0	6	HC	0	0	6	HC	2	0	7	Mean OA	std
	OA=	95,4			OA=	88,4			OA=	90,7			OA=	93,0			OA=	93,0		92,1	2,6533

test

	RA	PsA	HC		RA	PsA	HC		RA	PsA	HC		RA	PsA	HC		RA	PsA	HC		
RA	5	1	1	RA	4	2	3	RA	5	1	1	RA	5	0	0	RA	5	1	0		
PsA	1	4	0	PsA	1	3	0	PsA	1	5	0	PsA	1	5	1	PsA	1	5	1		
HC	0	1	3	HC	1	1	1	HC	0	0	3	HC	0	1	3	HC	0	0	3	Mean OA	std
	OA=	75,0			OA=	81,3			OA=	81,3			OA=	81,3			OA=	81,3		80,0	2,817446

biochemical and Raman datasets

CV

	RA	PsA	HC		RA	PsA	HC		RA	PsA	HC		RA	PsA	HC		RA	PsA	HC		
RA	19	0	0	RA	19	1	0	RA	20	1	0	RA	19	0	1	RA	19	0	0		
PsA	1	15	0	PsA	0	13	0	PsA	0	14	1	PsA	1	15	0	PsA	0	16	0		
HC	0	1	8	HC	1	2	8	HC	0	1	7	HC	0	1	7	HC	1	0	8	Mean OA	std
	OA=	95,5			OA=	97,7			OA=	93,2			OA=	93,2			OA=	97,7		95,5	2,250111

test

	RA	PsA	HC		RA	PsA	HC		RA	PsA	HC		RA	PsA	HC		RA	PsA	HC		
RA	5	1	0	RA	5	3	2	RA	6	1	1	RA	5	0	0	RA	6	1	0		
PsA	1	4	0	PsA	1	2	0	PsA	0	4	0	PsA	1	5	0	PsA	0	4	0		
HC	0	1	4	HC	0	1	2	HC	0	1	3	HC	0	1	4	HC	0	1	4	Mean OA	std
	OA=	81,3			OA=	93,8			OA=	81,3			OA=	87,5			OA=	87,5		86,3	5,223217

biochemical and NIR datasets

CV

	RA	PsA	HC		RA	PsA	HC		RA	PsA	HC		RA	PsA	HC		RA	PsA	HC		
RA	20	0	1	RA	17	1	0	RA	19	0	0	RA	19	0	1	RA	20	0	0		
PsA	1	14	0	PsA	2	12	0	PsA	1	15	0	PsA	2	14	0	PsA	0	15	1		
HC	0	1	6	HC	2	2	7	HC	1	0	7	HC	0	1	6	HC	1	0	6	Mean OA	std
	OA=	93,0			OA=	93,0			OA=	95,4			OA=	90,7			OA=	95,4		93,5	1,972308

test

	RA	PsA	HC		RA	PsA	HC		RA	PsA	HC		RA	PsA	HC		RA	PsA	HC		
RA	5	0	1	RA	5	2	2	RA	5	0	0	RA	6	1	1	RA	6	0	0		
PsA	1	5	0	PsA	0	1	0	PsA	0	5	0	PsA	0	4	0	PsA	0	5	1		
HC	0	1	3	HC	1	3	2	HC	1	1	4	HC	0	1	3	HC	0	1	3	Mean OA	std
	OA=	81,3			OA=	87,5			OA=	87,5			OA=	81,3			OA=	87,5		85,0	3,39588

Figure S14. Confusion matrices for CP-ANN models resulting from samples randomization (5 runs)
